# Supplementary material for: In Silico Analysis of miRNA-Mediated Genes in the Regulation of Dog Testes Development from Immature to Adult Form
Source: Animals (Basel). 2023 Apr 30;13(9):1520. doi: 10.3390/ani13091520 (PMC10177090; doi:10.3390/ani13091520)
Supplement: Supplementary file 1 [file animals-13-01520-s001.zip › animals-2307290-supplementary-S1.pdf]

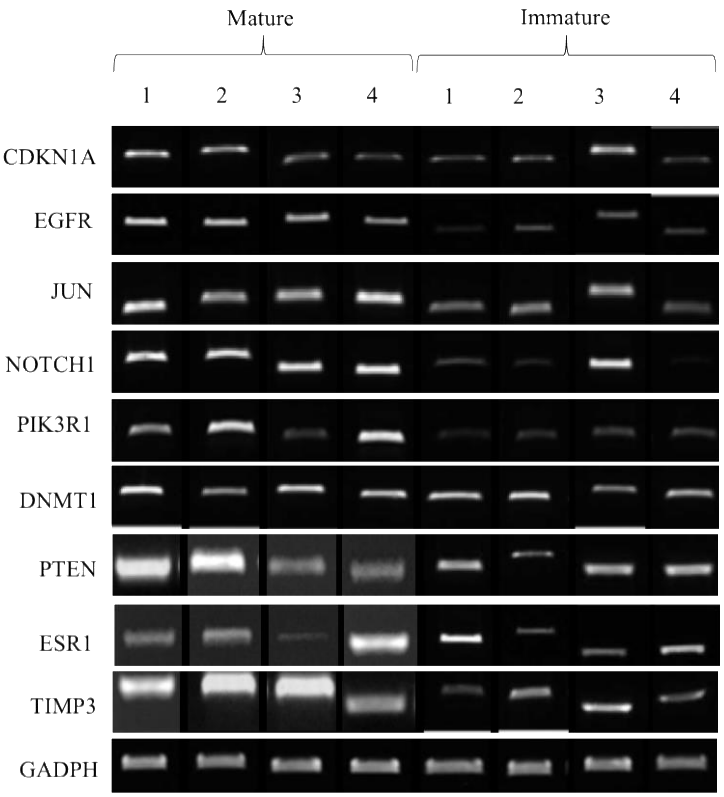

Supplementary file S1. The ethidium bromide-stained electrophoresis gel, with amplicons of expected sizes.

Expected amplicons from four mature and four immature dog testis for each gene target were shown.

CDKN1A, cyclin-dependent kinase inhibitor 1A, 18.1 kDa;
EGFR, epidermal growth factor receptor, ~180 kDa;
JUN, jun proto-oncogene, AP-1 transcription factor subunit, 43 kDa;
NOTCH1, notch receptor 1, 273 kDa;
DNMT1, DNA methyltransferase 1, ~180 kDa;
PIK3R1, phosphoinositide-3-kinase regulatory subunit 1, 85 kDa;
PTEN, phosphatase, and tensin homolog, 47 kDa;
ESR1, estrogen receptor 1, 66 kDa;
TIMP3, tissue inhibitor of metalloproteinases 3, 30 kDa;
GADPH, glyceraldehyde-3-phosphate dehydrogenase, 36 kDa;
