# Supplementary material for: In Silico Analysis of miRNA-Mediated Genes in the Regulation of Dog Testes Development from Immature to Adult Form
Source: Animals (Basel). 2023 Apr 30;13(9):1520. doi: 10.3390/ani13091520 (PMC10177090; doi:10.3390/ani13091520)
Supplement: Supplementary file 1 [file animals-13-01520-s001.zip › animals-2307290-supplementary-S8.pdf]

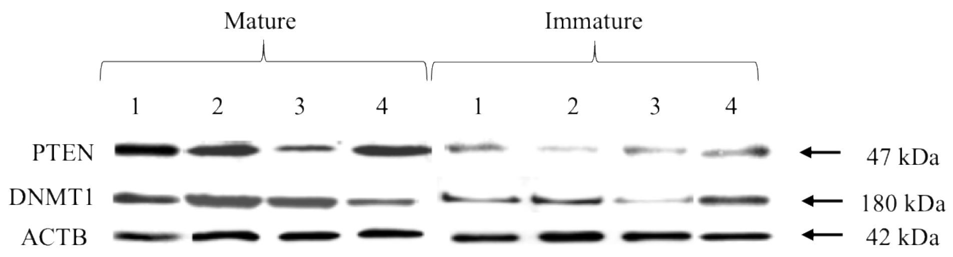

Supplementary File S8. Representative Western blots of isozymes .
PTEN, phosphatase and tensin homolog, DNMT1, dna methyltransferase 1; and ACTB, beta actin; PTEN, DNMAT1 and ACTB were 47, ~180 and 42 kDa, respectively.
